# Discovery of Potent Antimalarial Agents Targeting *Plasmodium falciparum* DNA Gyrase B by Integrating Computational and Experimental Approaches

**DOI:** 10.1111/cbdd.70364

**Published:** 2026-07-21

**Authors:** Biswajit Naik, Welka Sahu, Guneswar Sethi, Cherish Prashar, Gajendra Mohan Baldodiya, Jyoti Poswal, Chandi C. Mandal, Jeong Ho Hwang, Kailash C. Panday, K. Sony Reddy, Dhaneswar Prusty

**Affiliations:** ^1^ Department of Biochemistry School of Life Sciences, Central University of Rajasthan Ajmer Rajasthan India; ^2^ School of Biotechnology, Kalinga Institute of Industrial Technology Bhubaneswar Odisha India; ^3^ Animal Biotechnology and Genomics Division, National Institute of Animal Science, Rural Development Administration Jeonbuk State Republic of Korea; ^4^ ICMR‐National Institute of Malaria New Delhi India; ^5^ Academy of Scientific and Innovative Research (AcSIR) Gaziabad India; ^6^ Department of Animal Science and Technology, Konkuk University Seoul Republic of Korea

**Keywords:** antimalarial drug discovery, drug resistance, Gyrase B inhibitors, malaria, plasmodium falciparum

## Abstract

The global spread of drug‐resistant *Plasmodium falciparum*, particularly artemisinin‐resistant strains, underscores the urgent need for novel antimalarial agents with distinct mechanisms of action and improved therapeutic potential. In this study, we employed an integrated in silico and in vitro strategy to identify compounds with inhibitory activity against *P. falciparum* apicoplast Gyrase B (PfGyrB). High‐throughput virtual screening identified hit compounds with favorable predicted interactions against the target protein, which were subsequently evaluated using biochemical ATPase inhibition and parasite growth inhibition assays. UNC8153 and Fexofenadine hydrochloride demonstrated time‐dependent antiplasmodial activity, with lower IC_50_ values at 96 h than at 48 h under prolonged exposure conditions. UNC8153 exhibited greater antiplasmodial activity (~25‐fold) than the reference compound novobiocin during the second intraerythrocytic cycle. Morphological analysis indicated impaired parasite development during the second intraerythrocytic cycle under prolonged exposure conditions. Importantly, UNC8153 retained inhibitory activity against the artemisinin‐resistant C580Y strain and exhibited minimal cytotoxicity toward HEK‐293 cells under the tested experimental conditions. Structural similarity analysis indicated that the identified compounds are chemically distinct from currently used antimalarial drugs, supporting their structural novelty. Collectively, these findings identify UNC8153 as a structurally distinct new antiplasmodial scaffold warranting further mechanistic, pharmacological, and in vivo evaluation.

## Graphical abstract

An integrated computational and experimental drug‐discovery approach identified UNC8153 as a potential novel scaffold active against drug‐sensitive and artemisinin‐resistant *P. falciparum* strains through inhibition of apicoplast PfGyrB.

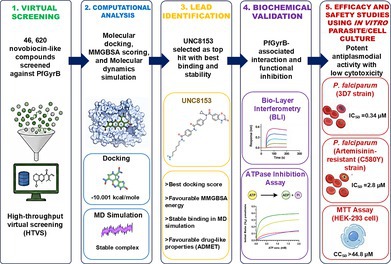

## 1 Introduction

Malaria continues to pose a significant global public health challenge. According to the World Malaria Report 2024, an estimated 263 million malaria cases and approximately 5,98,000 deaths were reported worldwide in 2023, representing an increase of about 14 million cases compared to 2022 (Venkatesan 2025). The absence of an effective vaccine and emerging cases of drug resistance are the major factors modulating this scenario (Siqueira‐Neto et al. 2023). *Plasmodium falciparum* is the deadliest species of Plasmodium, accounting for the majority of mortality. Currently, artemisinin‐based combination therapy is the only remedy to mitigate malaria. However, the emergence of partial artemisinin resistance highlights the urgent need for novel antimalarial agents with distinct mechanisms of action (Menard and Dondorp 2017).

The apicoplast, a plastid‐like organelle within the malaria parasite *Plasmodium falciparum* and related apicomplexans, is of immense interest as a drug target for malaria (Biddau and Sheiner 2019). Owing to its prokaryotic nature and evolutionary origin as a secondary endosymbiont, the apicoplast represents a unique site for novel drug intervention against the parasite (Janouskovec et al. 2010). This organelle is involved in type II fatty acid synthesis, isoprenoid biosynthesis, iron–sulfur cluster synthesis, and heme synthesis, all of which are essential for parasite survival. The apicoplast possesses a 35 kb genome, is self‐replicating, and performs housekeeping functions of prokaryotic origin. In the context of drug discovery, several antibiotics have been tested and shown to be antimalarials targeting apicoplast housekeeping functions (Botté et al. 2012).

A key drug target of apicoplast housekeeping function is DNA gyrase, which introduces negative supercoiling during DNA replication (M. A. Dar et al. 2007). Like bacterial DNA gyrase, it comprises a homodimer of the Gyrase subunits A and B. The Gyrase A subunit functions in DNA breakage and reunion, whereas Gyrase B provides the energy through its ATPase activity (Bopp et al. 2013). Despite many efforts to express the Gyrase A subunit, the Gyrase A protein has not been expressed to date. Whereas the full‐length PfGyrB was expressed, biochemically characterized, and proven as an excellent apicoplast drug target (M. A. Dar et al. 2007; Ram et al. 2007). The antibiotic novobiocin, which targets bacterial Gyrase B, has been shown to inhibit PfGyrB enzymatic activity and to possess antimalarial activity (Ram et al. 2007). Therefore, novobiocin and related compounds may act as antimalarials by primarily targeting PfGyrB. However, direct target engagement in vivo is unproven, and other targets may be involved in its mechanism of action.

This study focused on identifying novel antiplasmodial compounds with potential inhibitory activity against PfGyrB of the apicoplast. In this direction, we have opted for high‐throughput virtual screening, biochemical validation, and phenotypic screening to achieve the goal. We have screened 80% similar compounds to novobiocin against PfGyrB of the apicoplast. Based on docking and MMGBSA scores, we identified UNC8153 as the top‐ranked hit. Molecular dynamics simulations further supported stable interactions between UNC8153 and the PfGyrB binding site, while in silico drug‐likeness assessment suggested favorable physicochemical properties. Biolayer interferometry and ATPase inhibition assays demonstrated detectable interaction with PfGyrB and inhibition of PfGyrB ATPase activity under the tested experimental conditions. Phenotypic assays revealed that UNC8153 exhibited greater antiplasmodial activity than novobiocin and showed enhanced inhibitory effects during prolonged exposure against the drug‐sensitive *Plasmodium falciparum* 3D7 strain. In addition, UNC8153 retained activity against the artemisinin‐resistant C580Y parasite line at sub‐micromolar concentrations and displayed minimal cytotoxicity toward HEK‐293 cells under the tested conditions. Structural similarity analysis further indicated that UNC8153 is chemically distinct from currently used antimalarial agents. Collectively, these findings identify UNC8153 as a structurally distinct antiplasmodial scaffold associated with PfGyrB inhibition and support its further investigation through intracellular target‐validation studies, mechanistic characterization, pharmacokinetic evaluation, and in vivo efficacy assessment.

## 2 Materials and Methodology

### 2.1 Chemical Details

UNC8153 (company: ChemScene; catalog number: CS‐0908509, Purity (LCMS): 99.91%, Stock solution preparation: 10 mM in dimethyl sulfoxide (DMSO)), Fexofenadine hydrochloride (Company: ChemScene; catalog number: CS‐4483, Purity (LCMS): 99.86%, Stock solution preparation: 10 mM in DMSO), Novobiocin sodium salt (company: SRL; catalog number: 27434; Purity (HPLC): 95%, Stock solution preparation: 10 mM in deionized water), Malachite Green Phosphate Assay kit (Company: Sigma‐Aldrich; Catalog number: MAK307).

### 2.2 In Silico Study

#### 2.2.1 Homology Modeling of PfGyrB

The crystallographic structure of PfGyrB has not yet been determined. Hence, we performed homology modeling to get the nearest three‐dimensional structure. The homology modeling was performed by SWISS‐MODEL (https://swissmodel.expasy.org/), an automated protein structure homology modeling server. The amino acid sequences of each protein were retrieved from the PlasmoDB database (https://plasmodb.org/plasmo/app) and given as input to this server, and the final best quality models were chosen based on their QMEANDisCo scores. The modeled proteins were then refined using the 3D Refine server (https://3drefine.mu.hekademeia.org) and validated by plotting the Ramachandran plot and ProSA protein structure analysis server (https://prosa.services.came.sbg.ac.at/prosa.php).

#### 2.2.2 Designing the Compound Library for Screening

A Novobiocin‐like compound library was generated by selecting compounds with a Tanimoto structural similarity coefficient of ≥ 0.80. In total, 46,620 compounds were retrieved in SDF format from the PubChem (https://pubchem.ncbi.nlm.nih.gov/) and Chem Scene (https://www.chemscene.com/) databases.

#### 2.2.3 Virtual Screening

##### 2.2.3.1 Protein and Ligand Preparation

The protein PfGyrB was pre‐processed at physiological pH (pH 7.4) to mimic normal biological conditions (Ödinger Release 2021). This involved removing water molecules, filling missing side chains, and optimizing hydrogen bonding by adding hydrogen atoms. Energy minimization was subsequently carried out using the OPLS3 force field, all performed through the Protein Preparation Wizard module in Schrödinger (Jorgensen and Tirado‐Rives 1988). The 3D conformers of all the molecules were prepared using the OPLS‐2005 force field without changing their original state of charge in the Ligprep module of Schrödinger (LigPrep 2021).

##### 2.2.3.2 Grid Generation and Molecular Docking

The grid generation process was performed using GLIDE software's grid generation module to ensure precise ligand docking into the catalytic pocket (Friesner et al. 2006). A 20 Å grid box was created around the key active site residues (E159 and K220) of PfGyrB's ATPase domain to define the docking region. Molecular docking was subsequently conducted to assess the binding potential of the ligands to the target protein. A systematic screening approach was employed, beginning with High Throughput Virtual Screening (HTVS), followed by Standard Precision (SP) docking and Extra Precision (XP) docking with the Glide default settings, with 10% of compounds carried forward at each step. These progressively stringent docking protocols facilitated the identification of the most potent ligands based on their binding affinities within the catalytic pocket, providing a comprehensive evaluation of ligand‐protein interactions (Friesner et al. 2004).

##### 2.2.3.3 Binding Energy Estimation

Binding energy is a critical determinant of protein‐ligand interactions, as favorable binding energy indicates stronger and more stable interactions, while unfavorable energy can lead to the dissociation of the complex. The Molecular Mechanics Generalized Born Surface Area (MMGBSA) method was employed to evaluate the binding affinities of protein‐ligand complexes after the XP mode of docking. Binding energies, represented as MMGBSA scores in kcal/mol, were calculated to quantify interaction strength, with more negative scores denoting highly favorable interactions. The MMGBSA score was computed using the formula MMGBSA score = (Potential Energy of Complex) − (Potential Energy of Protein + Potential Energy of Ligand). The calculations were conducted using the Prime MMGBSA module of Schrödinger, employing the VSGB solvation model and the OPLS3 force field for optimal accuracy and reliability.

##### 2.2.3.4 ADMET Prediction

The ADMET (Absorption, Distribution, Metabolism, Excretion, and Toxicity) profiles of the top 10 compounds were systematically evaluated to characterize their drug‐like properties. The ADME parameters were predicted using the QikProp module of the Schrödinger suite, which provides insights into pharmacokinetic behavior and molecular properties (Schrödinger Release 2025–3: LigPrep). Toxicity profiles were assessed separately through the pkCSM online server (https://biosig.lab.uq.edu.au/pkcsm/) to identify potential safety concerns. These evaluations provided a comprehensive understanding of the pharmacological and toxicological characteristics of the selected compounds, facilitating their suitability for further investigation.

#### 2.2.4 Molecular Dynamics Simulation

A molecular dynamics simulation (MDS) was conducted to assess the stability of the protein‐ligand complex in a simulated biological setting. We used the Desmond module of Schrödinger for the simulation. To achieve this, a virtual dynamic environment was created with the help of a system builder panel using a single‐point charge water model surrounded by an orthorhombic boundary, neutralizing the system by adding Na^+^ and Cl^−^ ions and applying an OPLS 2005 force field (Bowers et al. 2006). The system was equilibrated at a constant temperature (300 K) and standard atmospheric pressure, i.e., 1 bar. The MDS was run for a 200‐ns (ns) time duration. After the simulation run time was completed, the trajectory file was examined to evaluate the conformational changes of the whole protein as Root mean square deviation (RMSD) and the conformational changes of individual amino acids as Root mean square fluctuation (RMSF). Both the RMSD and RMSF values were reported in angstrom (Å).

### 2.3 Protein Expression and Purification of PfGyrB

The plasmid for PfGyrB expression reported previously (Dar et al. 2007) was transformed into *Escherichia coli* strain BL21 Codon Plus. This plasmid includes the PfGyrB gene (Gene ID: PF3D7_1239500), lacking codons 1–200, cloned into the pET28a expression vector. The protein expressed from this construct corresponds to mature PfGyrB lacking the signal peptide and apicoplast‐targeting peptide and retains all functional domains required for enzymatic function. Transformed cells were cultured in 500 mL LB broth containing 25 μg/mL chloramphenicol and 50 μg/mL kanamycin, induced to an optical density of 0.4–0.6 with 0.5 mM IPTG (Isopropyl β‐D‐1‐thiogalactopyranoside) for 6 h at 28°C. After 6 h, cells were harvested by centrifugation at 8000 rpm for 10 min. Harvested cells were lysed in Lysis buffer (100 mM Na_2_HPO4, 100 mM NaH_2_PO4, 50 mM Tris‐Cl, pH 8.0, 300 mM NaCl, 10 mM β‐mercaptoethanol, 100 μM PMSF [Phenylmethylsulfonyl Fluoride]), recommended concentration of protease inhibitor cocktail, and 10 mM imidazole. The cells were lysed further by adding lysozyme at 1 mg/mL, incubating at 4°C for 1 h, followed by sonication (two rounds of 3 min at 32% amplitude) and centrifugation for 30 min at 11000 rpm. Total soluble proteins were then allowed to bind to Ni‐NTA (Nickel‐Nitrilotriacetic Acid) agarose resins for 1 h at 4°C. Protein‐bound resins were washed three times with wash buffer (100 mM Na_2_HPO4, 100 mM NaH_2_PO4, 50 mM Tris‐Cl, pH 8.0, 300 mM NaCl) containing 30 mM imidazole. His‐tagged protein was eluted in Elution buffer (100 mM Na_2_HPO4, 100 mM NaH_2_PO4, 50 mM Tris‐Cl, pH 8.0, 300 mM NaCl) containing 250 mM imidazole. Eluted protein was further dialyzed in a dialysis buffer containing 50 mM Tris‐Cl, pH 7.5, 100 mM KCl, 2 mM EDTA (Ethylenediaminetetraacetic Acid), 10 mM β‐mercaptoethanol, 100 μM PMSF, and 10% glycerol. The purity of the purified recombinant PfGyrB protein was assessed by 10% SDS‐PAGE (Sodium Dodecyl Sulfate‐Polyacrylamide Gel Electrophoresis) under reducing conditions. Protein bands were visualized by Coomassie Brilliant Blue staining, and the presence of a predominant band corresponding to the expected molecular weight of PfGyrB was confirmed. The oligomeric state of the purified protein was not experimentally determined in the present study.

### 2.4 Bio‐Layer Interferometry

Bio‐layer interferometry (BLI), a label‐free technique (Concepcion et al. 2009) for analyzing real‐time biomolecular interactions, was employed to investigate the binding interactions between PfGyrB and the top hit compounds, UNC8153 and Fexofenadine hydrochloride, along with the control drug Novobiocin. UNC8153, Fexofenadine hydrochloride, and Novobiocin were prepared at varying concentrations using PBS buffer (pH 7.4) containing 1% DMSO. The purified His‐tagged PfGyrB was immobilized on nickel‐nitrilotriacetic acid‐coated biosensors (OCTET, SARTORIUS) to facilitate interaction studies. The biosensors underwent a washing and baseline step for 60 s in PBS buffer with 1% DMSO. The biosensors were then immersed in wells containing different concentrations of UNC8153 (25, 50,100, and 200 μM), Novobiocin (25, 50,100, and 200 μM), and Fexofenadine hydrochloride (50,100, 200, and 400 μM) for an association phase of 600 s, followed by a dissociation phase of 600 s. The binding kinetics were analyzed using Octet Data Analysis Software, which fit the sensorgrams to a 1:1 binding model, indicating the interaction of a single ligand with a single binding site on the protein. The dissociation constant (*K*_D_) was calculated by taking the ratio of the dissociation rate constant (*k*_off_, s^−1^) to the association rate constant (*k*_on_, M^−1^·s^−1^), according to the formula: *K*_D_ = *k*_off_/*k*_on_. The equilibrium dissociation constant (*K*_D_) provides insights into the binding affinity between PfGyrB‐UNC8153, PfGyrB‐Fexofenadine hydrochloride, and PfGyrB‐Novobiocin.

### 2.5 ATPase Inhibition Assay

The substrate‐dependent ATPase assay was measured using a Malachite green phosphate assay Kit purchased from Sigma. The ELISA plate reader estimated the release of Phosphate during the hydrolysis of ATP by PfGyrB at 620 nm. Reactions with 80 μL mixtures were carried out at room temperature in a reaction buffer containing 35 mM Tris‐Cl (pH 7.5), 70 mM KCl, 5 mM MgCl_2_, 1 μM of PfGyrB protein, dsDNA (15 μg/mL), various concentrations of ATP (0.05, 0.1, 0.15, 0.2, 0.25, 0.5, 0.75, 1, 1.5, 2 mM), incubated for 30 min. After that, 20 μL of the Malachite green working reagent was added, and the absorbance was measured at 620 nm. The rate of ATP hydrolysis was determined after subtracting the control reaction rate lacking the protein PfGyrB. We treated the reaction mixture with 10 μM concentrations of UNC8153, Fexofenadine hydrochloride, and Novobiocin separately to investigate the inhibition of PfGyrB ATPase activity. The *V*_max_ and *K*_m_ were calculated using the non‐linear Michaelis–Menten model, and *K*_i_ was determined for competitive inhibition using the non‐linear competitive inhibition Model of GraphPad Prism v9.3.1.

### 2.6 Parasite Growth Inhibition Assay in Drug‐Sensitive and Artemisinin‐Resistant Strains

The blood‐stage Plasmodium parasites (*Plasmodium falciparum* 3D7 and *Plasmodium falciparum* C580Y) were cultured in vitro according to the standard protocol (M. A. Dar et al. 2007), using O‐positive human erythrocytes from voluntary donors collected in EDTA‐coated sterile tubes. A 5% sorbitol solution was employed at regular intervals to synchronize the parasites. To evaluate the effects of UNC8153 and Novobiocin against *Plasmodium falciparum* 3D7, various drug concentrations were administered to parasite cultures with an initial parasitaemia of 0.5% in a 96‐well plate. Note that parasites were continuously exposed to the test compound, which differs from the conventional procedure for assessing delayed death in which parasites are exposed for 48 h and enumerated after progression through the second cycle in the absence of the test compound (Dahl and Rosenthal 2007; Yeh and DeRisi 2011). Therefore, alternative methods are required to validate compounds with delayed death mechanisms of action for the procedure we opted for in this study. Parasites were harvested in different stages (first and second life cycles) by replacing the culture medium every 24 h to ensure consistent nutrient availability. Thin blood smears were stained with Giemsa, and at least 1000 erythrocytes were examined microscopically. Parasitemia was calculated as the percentage of morphologically normal infected erythrocytes among total erythrocytes counted. Pyknotic parasites, abnormal forms, and arrested developmental stages were excluded from parasitemia determination. We have evaluated the efficacy of UNC8153 in the artemisinin‐resistant strain of *Plasmodium falciparum* (C580Y) using the same method. For this, the artemisinin‐resistant *Plasmodium falciparum* strain (CamWT_C580Y) carrying the K13‐C580Y mutation was originally obtained from the MR4 (Malaria Resources), a widely recognized source of authenticated malaria parasite strains. Parasites were harvested at different stages (first and second life cycles), and parasitaemia levels were quantified by microscopic examination. This experiment was performed in triplicate to ensure reproducibility and reliability of the results.

### 2.7 MTT Assay

The MTT assay, using 3‐(4,5‐dimethylthiazol‐2‐yl)‐2,5‐diphenyltetrazolium bromide (MTT), is a widely used colorimetric method for assessing cell viability and proliferation in cell culture models (Adan et al. 2016). In this study, the HEK‐293 (Human Embryonic Kidney 293 Cells) cell line was used to evaluate the effects of the compound UNC8153 on cell viability and proliferation. HEK‐293 cells were cultured in DMEM (Dulbecco's Modified Eagle Medium) medium supplemented with 10% fetal bovine serum (FBS). The cells were seeded in 96‐well plates at a density of 1 × 10^4^ cells per well. Different concentrations of UNC8153 (0.087, 0.175, 0.35, 0.7, 1.4, 2.8, 5.6, 11.2, 22.4, 44.8 μM) and Fexofenadine hydrochloride (1.87, 3.75, 7.5, 15, 30, 60, 120, and 240 μM) were added to each well, with a final volume of 100 μL per well consisting of DMEM, 3% FBS, and 1% antibiotics (penicillin and streptomycin). After 24 h of incubation, 10 μL of MTT solution (5 mg/mL in PBS) was introduced to each well, followed by an additional 2‐h incubation at 37°C. After removing the culture medium, 200 μL of DMSO was added to each well to dissolve the formazan crystals, and the plate was incubated at room temperature for 10 min. The absorbance was then measured at 530 nm on an ELISA plate reader after 2 min of orbital shaking. The assay was performed in triplicate to ensure consistent results.

### 2.8 Structural Similarity Determination Between UNC8153, Fexofenadine Hydrochloride and Existing Antimalarial Drugs

Exploring novel pharmacological scaffolds with diverse structural similarities to existing antimalarials is crucial to combating drug resistance. We used the ChemMine Tool (https://chemminetools.ucr.edu/) to determine the Tanimoto coefficient (Tc) similarity index and to create a hierarchical clustering based on the distance matrix to compare the structural similarities between the tested compounds (UNC8153 and Fexofenadine hydrochloride) and well‐known antimalarial compounds. The Tanimoto coefficient ranges from 0 to 1, with higher values indicating greater structural similarity. A Tanimoto coefficient value greater than 0.85 indicates a significant degree of drug similarity (Corey et al. 2016). Furthermore, the distance matrix score was calculated as 1‐Tanimoto coefficient, and its value ranges from 0 to 1, with lower values indicating stronger structural similarity (Matter 1997). Using the distance matrix score, a hierarchical clustering of all compounds was performed to create a phylogenetic structural similarity tree with the hclust script. We have selected six antimalarial compounds from diverse classes, including artemisinin, chloroquine, tetracycline, proguanil, sulfadoxine, and pyrimethamine, to investigate structural similarities (Travassos and Laufer 2017).

## 3 Results

### 3.1 Homology Modeling and Model Validation of PfGyrB

**FIGURE 1:**
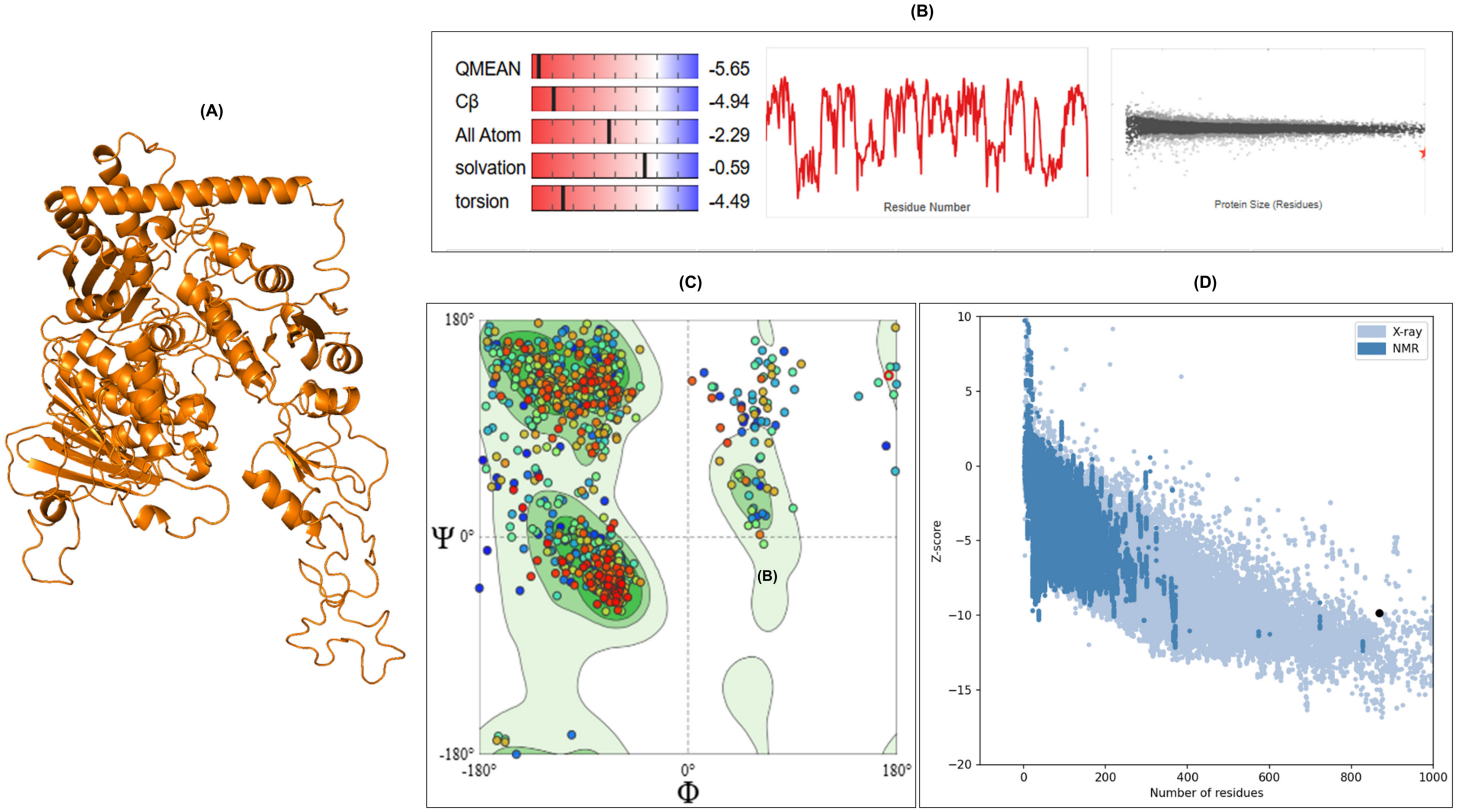
Illustration of (A) 3D modeled protein structure of PfGyrB, (B) residue‐wise local QMEAN score plot showing regional model confidence, (C) Ramachandran plot, and (D) ProSA *Z* score plot.

As the 3D crystallographic structure of PfGyrB is unavailable, we generated a homology model of this protein using SWISS‐MODEL. The homology model PfGyrB was developed by using the template 6gav.1.A of *Mycobacterium tuberculosis* , with 0.4 sequence similarity, 39.82 sequence identity, and 0.66 coverage. The QMEANDisCo score, which represents the model protein's quality, was used to select the best model for each protein. A model's QMEANDisCo score ranges between 0 and 1, with values approaching 1 indicating a high‐quality model (Benkert et al. 2010; Naik and Prusty 2024). The QMEANDisCo score for the modeled protein PfGyrB was 0.68 ± 0.05, exceeding 0.5, indicating a high‐quality model. Again, Residue‐wise local QMEAN analysis indicated that most regions of the PfGyrB homology model exhibited acceptable confidence scores, although increased fluctuations were observed in selected loop regions, suggesting comparatively lower local structural confidence. The modeled protein was subsequently optimized to reach a minimum energy state. Its 3D conformation was verified by generating a Ramachandran plot, and finally, the overall model quality was estimated using a *Z*‐score. From the Ramachandran plot of the modeled protein, the percentage of amino acids found in the favored conformational region was assessed. By ProSA, the overall model quality was estimated as a *Z*‐score by comparing the existing crystallographic structure solved by X‐ray and NMR (Wiederstein and Sippl 2007). The Ramachandran plot of the refined PfGyrB model revealed that 94.23% of residues lie in the favored region. The *Z*‐score of the modeled protein PfGyrB was estimated as −9.86, which falls within the acceptable ideal *Z*‐score range of 10 to −17 (Naik et al. 2024; Sippl 1993). This critical evaluation showed that the crystallographic and homology‐modeled protein is suitable for further docking studies. The 3D protein structure, residue‐wise local QMEAN score plot showing regional model confidence, the Ramachandran plot, and the ProSA model quality of PfGyrB are illustrated in Figure 1.

### 3.2 Molecular Docking, Binding Energy Estimation, MD Simulation Study, and ADMET Analysis Reveal Potential Hit Compounds Targeting PfGyrB

**FIGURE 2:**
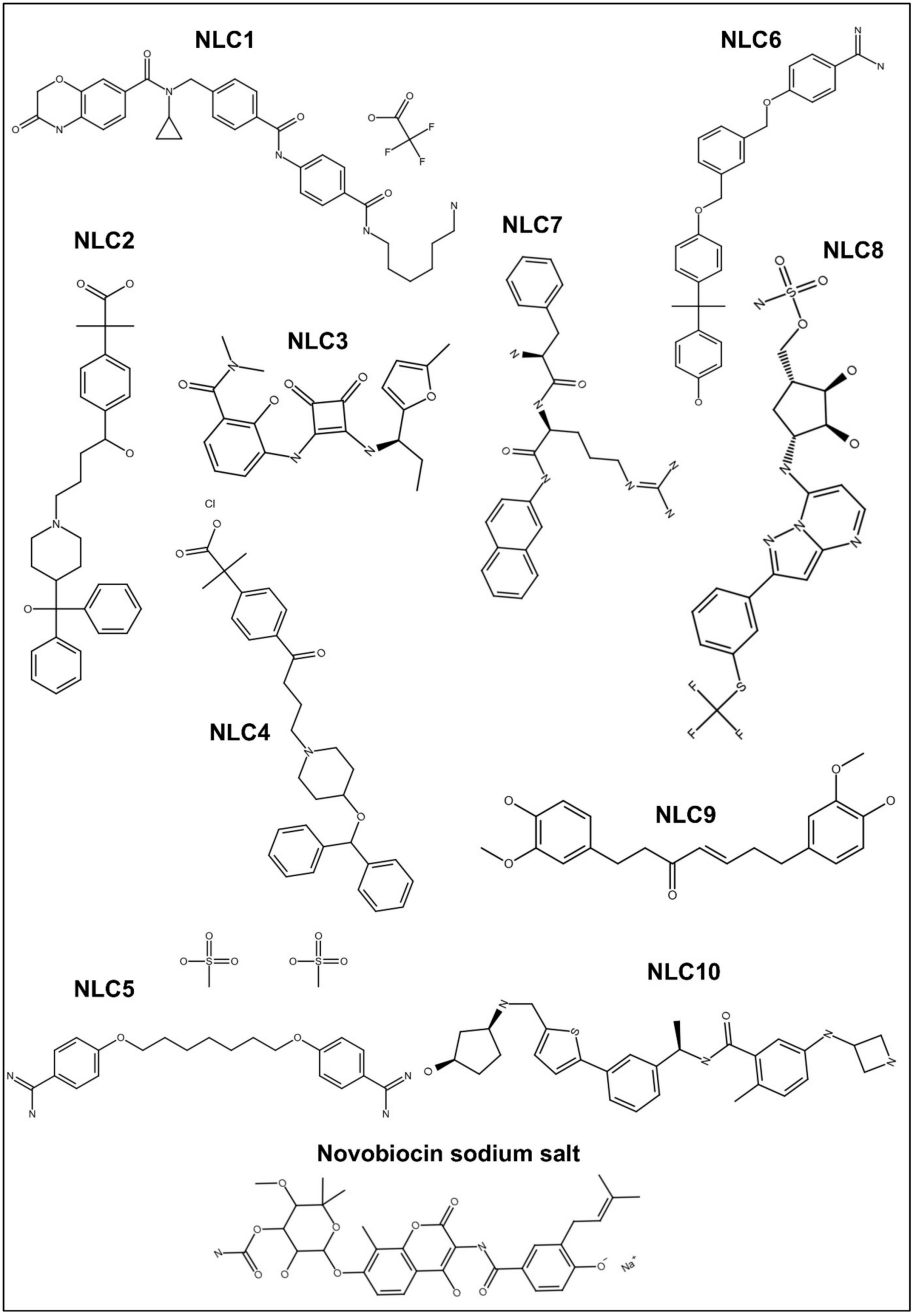
Chemical structures of the top 10 screened compounds and the reference compound novobiocin sodium salt (SRL catalog no: 27434).

In this study, we have curated a compound library 80% similar to Novobiocin (46,620 compounds) from the PubChem and ChemScene databases and performed structure‐based virtual screening using the Glide docking protocol to identify potent analogs targeting the ATP‐binding pocket of PfGyrB. The docking and MMGBSA scores of the best ten NLCs and the control Novobiocin are depicted in Table 1, and their 2D structures are illustrated in Figure 2.

**TABLE 1:** Molecular docking and MMGBSA binding energy analysis of the top 10 screened NLC compounds and the reference inhibitor Novobiocin against *Plasmodium falciparum* Gyrase B (PfGyrB).

Compound name	PubChem ID	Docking score (kcal/mol)	MMGBSA dG Bind (kcal/mol)
NLC1	169492943	−10.001	−83.54
NLC2	63002	−8.394	−73.52
NLC3	9865554	−7.045	−66.09
NLC4	65820	−6.929	−70.67
NLC5	146047121	−6.822	−64.97
NLC6	23292598	−6.739	−64.23
NLC7	90665180	−6.618	−64.58
NLC8	71715374	−6.445	−69.17
NLC9	5281775	−6.35	−63.04
NLC10	155801617	−6.042	−68.62
Novobiocin	54675769	−5.627	−62.42

**FIGURE 3:**
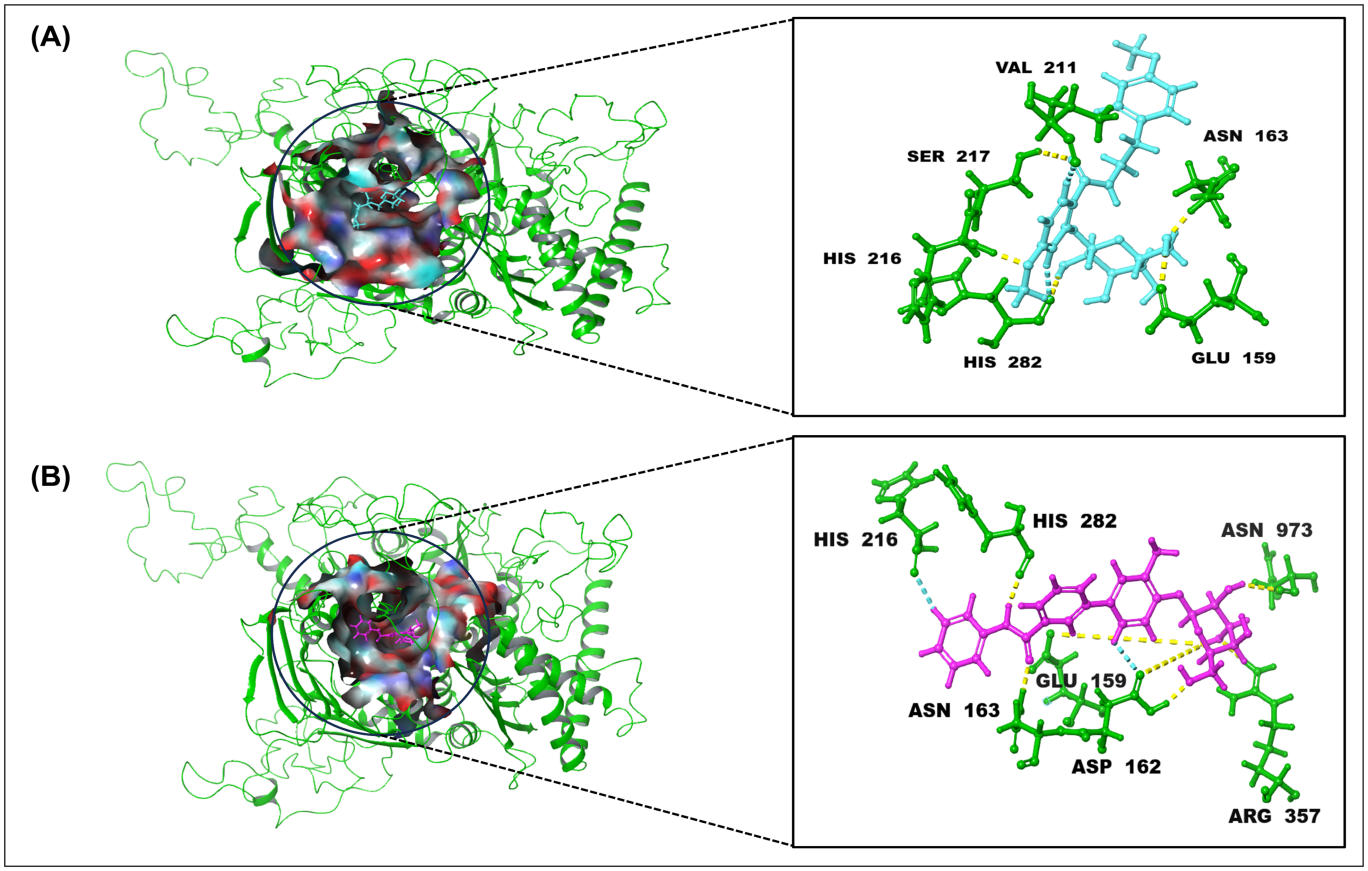
Illustration of the binding mode of (A) NLC1 (UNC8153) and (B) NLC2 (Fexofenadine hydrochloride) with PfGyrB and their interactions. Yellow and cyan color dotted lines represent the hydrogen and pi‐stacking interactions, respectively.

Further, we investigated whether the lead molecules interact with the active site pocket of PfGyrB. We analysed an atomistic binding interaction study of the top two NBDs, i.e., NLC1 (Commercial name: UNC8153) and NLC2 (Commercial name: Fexofenadine hydrochloride), with PfGyrB. The docked complex of NLC1 and NLC2 with PfGyrB shows that both bind the ATPase domain of PfGyrB via hydrogen bonds and pi‐stacking interactions. NLC1 shows 1.78‐ and 1.34‐fold higher docking and MMGBSA scores than control Novobiocin, forming hydrogen bonds with amino acid residues GLU159, ASN163, VAL211, HIS216, SER217, HIS282, and pi‐pi stacking with VAL211 and HIS282 of the PfGyrB active site pocket (Figure 3A). On the other hand, the NCL2 showed a 1.49‐ and 1.18‐fold higher docking and MMGBSA score than Novobiocin, making hydrogen bonds with GLU159, ASP162, ASN163, HIS282, ARG357, ASN973, and pi‐pi stacking interaction with ASP162 and HIS216 of the PfGyrB active site pocket (Figure 3B). It is worth mentioning that both NLC1 and NLC2 have been shown to form a strong hydrogen bond with the essential conserved amino acid GLU159 (E159), which might potentially limit PfGyrB's ATPase activity.

**FIGURE 4:**
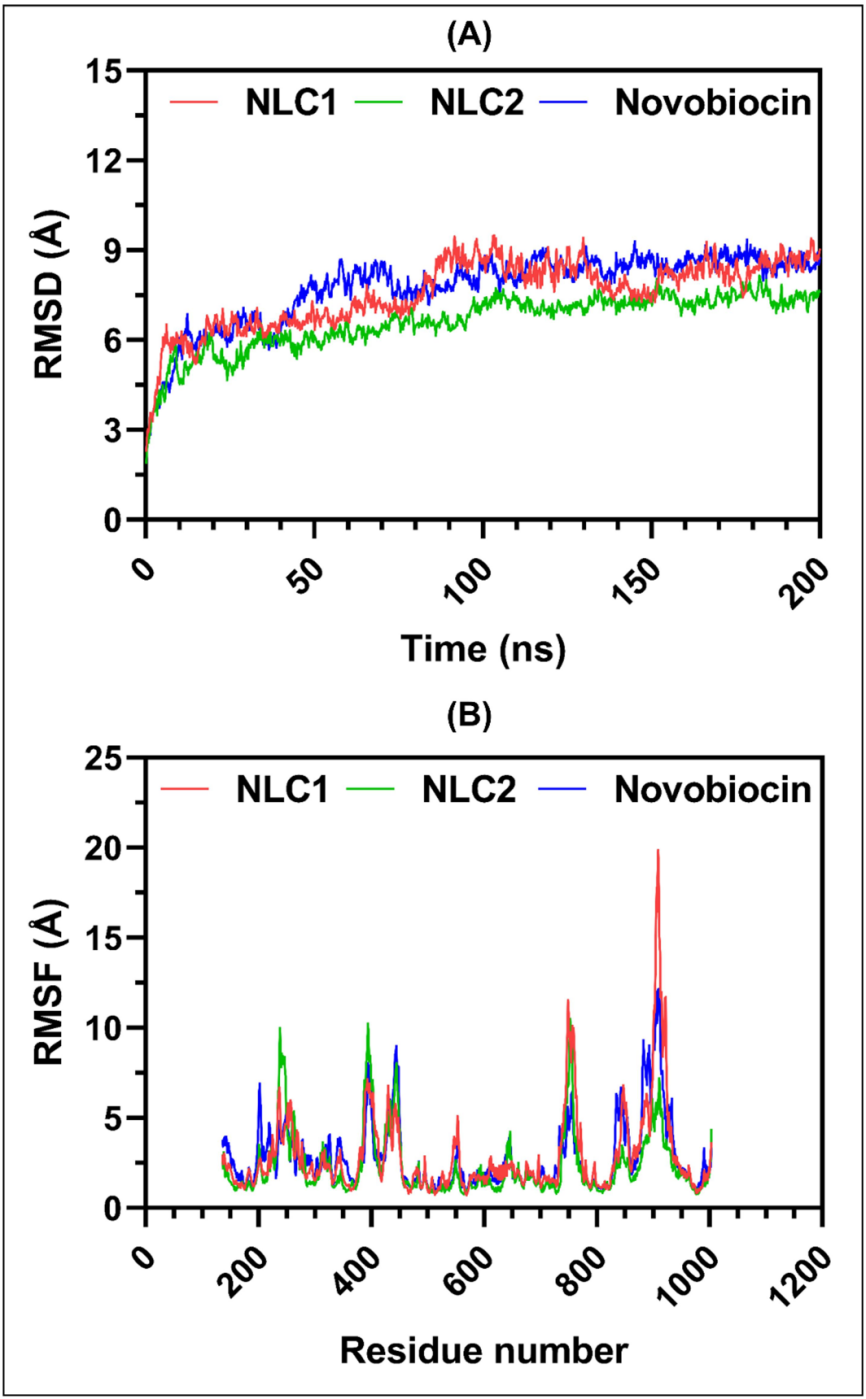
Illustration of molecular dynamics simulation of NLC1 (UNC8153) and NLC2 (Fexofenadine hydrochloride) in complex with PfGyrB, representing (A) protein RMSD plot, and (B) protein RMSF plot.

To assess the structural stability of the complexes, 200 ns Molecular Dynamics Simulations (MDS) were conducted. RMSD analysis of PfGyrB (Figure 4A) revealed an average backbone fluctuation of ~7.57 Å for NLC1, ~6.62 Å for NLC2, and ~7.75 Å for Novobiocin, with stable structural fluctuation throughout the simulation period. The RMSF analysis of PfGyrB in response to NLC1, NLC2, and Novobiocin (Figure 4B) indicated localized flexibility, with significant residue fluctuations (up to 20 Å) near loop regions. However, the core residues within the active site remained relatively stable, confirming strong and persistent interactions between PfGyrB and the analogs. Together, these findings demonstrate that NLC1 (UNC8153) and NLC2 (Fexofenadine hydrochloride) are potent and stable binders of PfGyrB, showing improved binding energetics and dynamic behavior over Novobiocin. These ligands serve as promising hits for future antimalarial drug development targeting apicoplast Gyrase B.

Furthermore, the physicochemical descriptors and drug‐like properties of the top 10 screened hits, together with the reference compound novobiocin, are summarized in Table 2. The predicted ADME properties are tabulated in Table 3, whereas the toxicity‐related parameters are shown in Table 4. Most compounds generally complied with the commonly accepted ranges for major physicochemical descriptors such as molecular weight, lipophilicity, hydrogen bond donors/acceptors, and predicted toxicity profiles. However, selected compounds showed minor deviations from recommended ranges for certain parameters. For example, UNC8153 (NLC1) exhibited a slightly elevated solvent‐accessible surface area (SASA; 1032.46 Å^2^) relative to the commonly accepted range (300–1000 Å^2^), while NLC2, NLC4, NLC6, and Novobiocin showed lower predicted aqueous solubility (QPlogS) values than the preferred threshold. Predictive absorption analysis indicated variable Caco‐2 permeability among the identified compounds. Compounds such as NLC2, NLC4, NLC6, NLC9, and NLC10 exhibited comparatively higher predicted permeability values, suggesting improved intestinal absorption potential, whereas NLC1 and NLC5 showed relatively lower predicted permeability. Despite this, most compounds demonstrated acceptable overall physicochemical properties supportive of oral drug‐like behavior. BBB permeability (logBB) and CNS permeability (logPS) predictions suggested generally low central nervous system penetration for most compounds. The observed logBB values remained below the threshold typically associated with efficient blood–brain barrier crossing, while the logPS values were largely consistent with limited CNS exposure. These findings may indicate reduced probability of CNS‐associated adverse effects, although experimental pharmacokinetic validation will be necessary. Metabolism and toxicity prediction analyses further indicated that most compounds were predicted to be non‐substrates and non‐inhibitors of CYP2D6, except NLC7 and NLC10, which showed predicted CYP2D6 inhibitory properties. Importantly, none of the compounds were predicted to exhibit AMES toxicity, hERG‐I inhibition, renal OCT2 substrate activity, or skin sensitization. Collectively, these results suggest generally acceptable in silico pharmacokinetic and toxicity profiles for the identified compounds while also highlighting selected parameters that may require further optimization and experimental validation.

**TABLE 2:** Predicted physicochemical descriptors and drug‐like properties of the top 10 screened lead compounds against PfGyrB.

Compound name	Mol WT (130–750)	SASA (300–1000)	Log Po/w (−2 to 6.5)	QPlogS (−6.5 to 0.5)	QPlogKhsa (−1.5 to 1.5)	Donor HB (0–6)	Accept HB (2–20)
NLC1	583.69	1032.46	3.29	−6.51	0.40	5.00	12.25
NLC2	501.66	890.66	4.14	−7.26	1.01	3.00	6.45
NLC3	397.43	744.04	2.38	−5.13	−0.03	2.00	9.25
NLC4	499.65	913.22	4.31	−7.59	0.99	1.00	7.70
NLC5	368.48	764.20	3.28	−4.77	0.03	6.00	4.50
NLC6	466.58	862.46	6.19	−7.91	1.22	4.00	3.75
NLC7	446.55	767.84	1.49	−2.50	−0.27	7.25	7.25
NLC8	519.51	788.97	2.12	−5.88	−0.15	5.00	10.40
NLC9	356.42	687.47	3.78	−5.06	0.39	2.00	5.00
NLC10	504.69	914.27	5.00	−7.12	1.05	5.00	6.70
Novobiocin	612.63	990.92	2.65	−7.25	0.28	5.25	13.15

**TABLE 3:** Predicted ADME profiles of the top 10 screened lead compounds and the reference compound Novobiocin.

Compound name	Absorption	Distribution		Metabolism		Excretion	
	Caco2 permeability (log Papp in 10^−6^ cm/s)	BBB permeability (Log BB)	CNS permeability (Log PS)	CYP2D6 Substrate (Yes/No)	CYP2D6 inhibitor (Yes/No)	Total Clearance (Log ml/min/kg)	Renal OCT2 substrate (Yes/No)
NLC1	−0.71	−1.593	−3.353	No	No	0.588	No
NLC2	0.753	−0.894	−0.894	No	No	0.827	No
NLC3	−0.097	−0.997	−2.694	No	No	0.047	No
NLC4	0.487	−0.361	−2.09	No	No	0.813	No
NLC5	−1.294	−2.059	−3.945	No	No	0.227	No
NLC6	0.662	−1.027	−1.928	No	No	−0.698	No
NLC7	0.312	−0.657	−3.014	No	Yes	0.873	No
NLC8	0.443	−1.991	−3.715	No	No	0.225	No
NLC9	0.98	−0.366	−2.566	No	No	0.205	No
NLC10	1.071	−0.709	−2.402	No	Yes	1.385	No
Novobiocin	0.183	−1.905	−3.459	No	No	−0.452	No

**TABLE 4:** Toxicity profile of the top 10 lead compounds.

Compound name	AMES toxicity	Max. tolerated dose (human)	hERG I inhibitor	Oral Rat Acute Toxicity (LD50)	Skin Sensitisation	Minnow toxicity
	(Yes/No)	(log mg/kg/day)	(Yes/No)	(mol/kg)	(Yes/No)	(log mM)
NLC1	No	0.293	No	2.388	No	1.378
NLC2	No	0.449	No	2.743	No	0.598
NLC3	No	0.253	No	2.394	No	3.209
NLC4	No	0.728	No	2.467	No	−0.696
NLC5	No	0.226	No	2.434	No	0.516
NLC6	No	0.038	No	2.709	No	−1.862
NLC7	No	−0.053	No	2.48	No	4.213
NLC8	No	0.536	No	2.219	No	2.947
NLC9	No	0.159	No	1.894	No	−0.539
NLC10	No	0.262	No	2.571	No	4.517
Novobiocin	No	0.475	No	2.714	No	3.902

### 3.3 Bio‐Layer Interferometry Assay Reveals UNC8153 Binds With PfGyrB, Similar to Novobiocin

**FIGURE 5:**
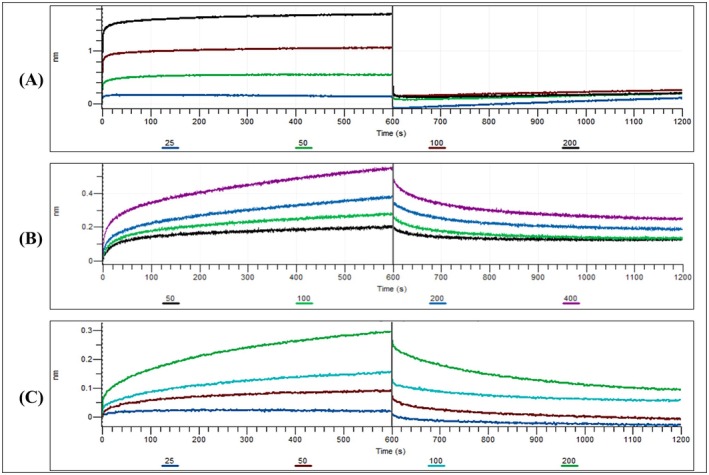
Real‐time Bio‐Layer Interferometry (BLI) sensorgrams illustrating the binding kinetics of small‐molecule inhibitors with recombinant PfGyrB. (A) Sensorgrams showing association and dissociation of UNC8153 with PfGyrB at concentrations of 25 μM (blue), 50 μM (green), 100 μM (red), and 200 μM (black). (B) Binding curves for Fexofenadine hydrochloride at 50 μM (blue), 100 μM (green), 200 μM (cyan), and 400 μM (purple). (C) Sensorgrams for Novobiocin, a known GyrB inhibitor, at 25 μM (blue), 50 μM (maroon), 100 μM (cyan), and 200 μM (green). In each plot, the *X*‐axis represents time in seconds, with association from 0 to 600 s and dissociation from 600 to 1200 s. The *Y*‐axis indicates binding response in nanometers (nm), reflecting the change in optical thickness upon ligand binding to immobilized PfGyrB.

The purified PfGyrB protein analysed through SDS‐PAGE (Supplementary Figure S1) was immobilized on the Ni‐NTA biosensor, which was then exposed to various concentrations of UNC8153 (25, 50, 100, 200 μM), Fexofenadine hydrochloride (50,100, 200, 400 μM), and the known drug Novobiocin (25, 50, 100, 200 μM), allowing for real‐time calculation of association rate (*k*_on_), dissociation (*k*_off_) rate, and dissociation constant (*K*_D_). The result reveals that the UNC8153 and Novobiocin showed a similar binding affinity with PfGyrB, with *K*_D_ values of 489.9 and 494.5 μM, respectively (Figure 5). The drug Fexofenadine hydrochloride showed a *K*_D_ value of 619.6 μM, suggesting a lower binding affinity with PfGyrB. The *K*_D_ values are calculated using the 1:1 global binding interaction model with correlation (*R* ^2^) values of 0.91, 0.94, and 0.93 for UNC8153, Fexofenadine hydrochloride, and Novobiocin, respectively. In this study, BLI measurements were performed without replicate‐derived kinetic error estimation, and the UNC8153 sensorgram exhibited atypical association‐dissociation behavior; these kinetic parameters should be interpreted cautiously and considered supportive evidence of interaction rather than definitive quantitative affinity measurements.

### 3.4 UNC8153 and Novobiocin Show Comparable ATPase Inhibition of PfGyrB

**FIGURE 6:**
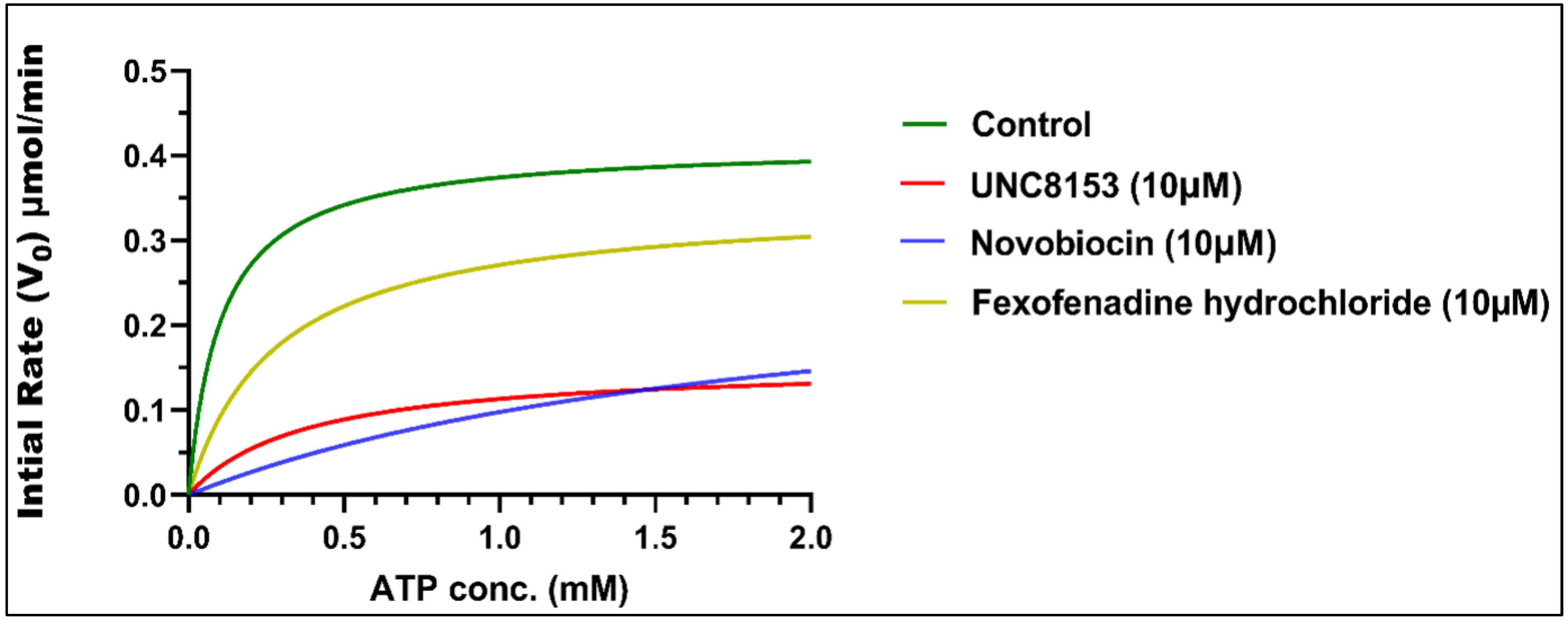
ATPase inhibition assay of recombinant PfGyrB in the presence of UNC8153, novobiocin, and Fexofenadine hydrochloride. Michaelis–Menten plots were generated by measuring the initial ATP hydrolysis rate (*V*_0_) at increasing ATP concentrations in the absence (control) or presence of 10 μM test compounds. Curves represent nonlinear regression fits to the Michaelis–Menten equation. The green curve corresponds to the untreated control, whereas the red, blue, and yellow curves represent UNC8153 (10 μM), novobiocin (10 μM), and Fexofenadine hydrochloride (10 μM), respectively. The *x*‐axis indicates ATP concentration (mM), and the y‐axis represents the initial reaction velocity (*V*_0_, μmol/min). Data were obtained from two independent experiments (*n* = 2).

To evaluate the inhibitory potential of selected hit compounds against *Plasmodium falciparum* Gyrase B (PfGyrB), an ATPase inhibition assay was performed using the malachite green phosphate detection method. The DNA‐dependent ATPase activity was assessed in the presence of 10 μM of the test compounds UNC8153, Fexofenadine hydrochloride, and the known drug Novobiocin in 2 biological independent experiment with triplicate measurements (Figure 6). The release of free phosphate was monitored, and the kinetic parameters, including the initial velocity (*V*_0_), maximum velocity (*V*_max_), Michaelis–Menten constant (*K*_m_), and inhibition constant (*K*ᵢ), were calculated under competitive inhibition conditions to determine the efficacy of each inhibitor (Mean ± SEM). The *V*_max_ and *k*_m_ were calculated as 0.416 ± 0.014 and 0.110 ± 0.022 for control (*R* ^2^ = 0.89). Subsequently, the inhibition constant (*K*ᵢ) for competitive inhibition was estimated using the competitive inhibition model of GraphPad Prism software. The compounds UNC8153 and Novobiocin demonstrated comparable inhibitory effects on PfGyrB, with Kᵢ values of 0.393 ± 0.044 μM and 0.320 ± 0.023 μM, respectively, indicating strong and similar ATPase inhibition profiles. In contrast, Fexofenadine hydrochloride exhibited a significantly higher *K*ᵢ value of 2.92 ± 0.84 μM, suggesting a weaker inhibition of the ATPase activity. These results reinforce the findings from the BLI analysis and support further investigation of UNC8153 as a potential PfGyrB‐associated antiplasmodial scaffold. The raw data from two biological experiments, including the initial rate (*V*_o_) under varying ATP concentrations, are given in Table S1.

### 3.5 Dose‐Dependent Inhibition of *Plasmodium falciparum* Growth by UNC8153, Fexofenadine Hydrochloride, and Novobiocin

**FIGURE 7:**
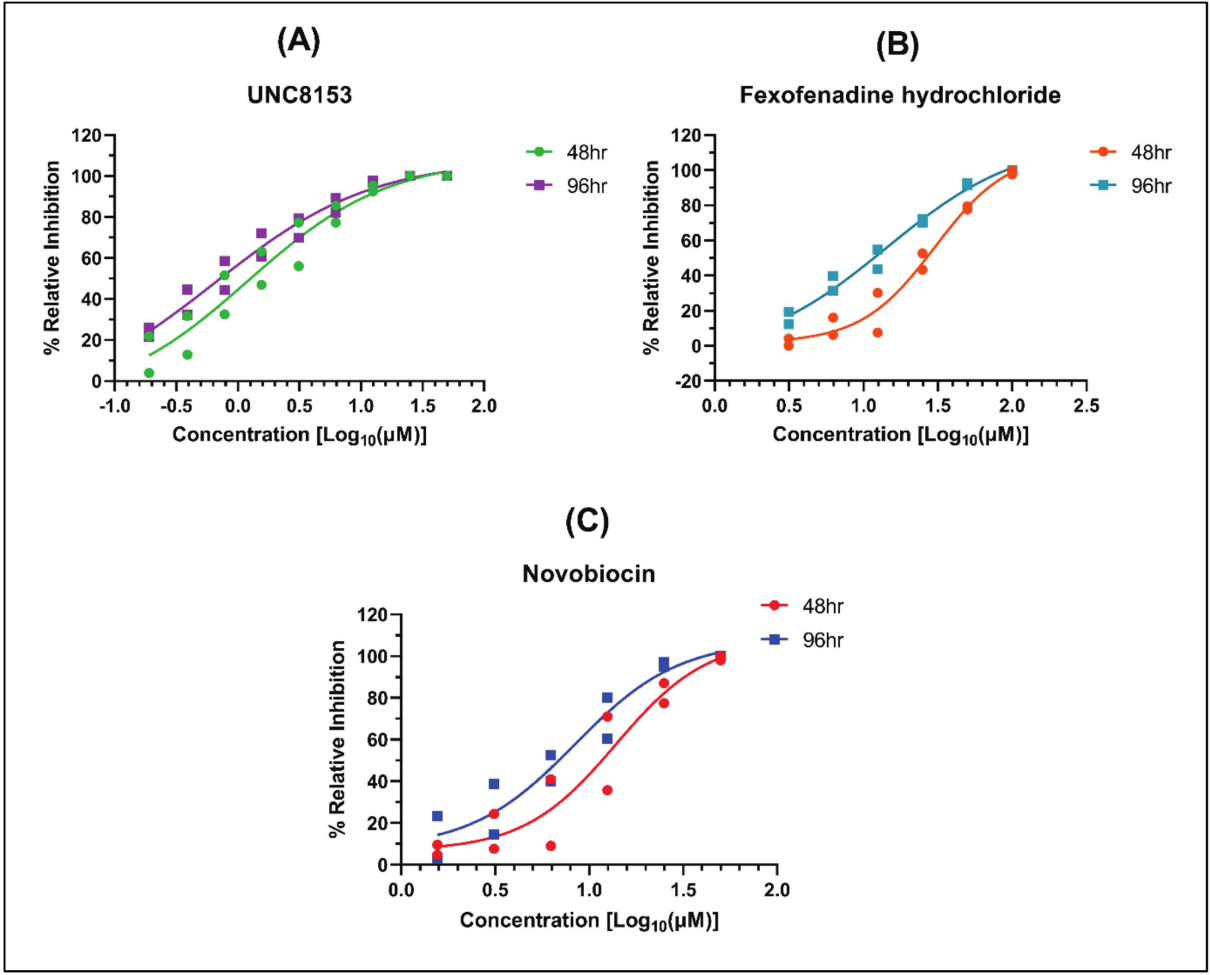
Dose–response analysis of parasite growth inhibition following treatment with (A) UNC8153, (B) Fexofenadine hydrochloride, and (C) novobiocin under continuous exposure conditions. The percentage relative inhibition of *Plasmodium falciparum* growth was determined after 48 h and 96 h of treatment. Curves were generated using a four‐parameter variable‐slope nonlinear regression model in GraphPad Prism for IC_50_ determination. Hill slope values obtained from the fitted models were 0.584 (48 h) and 0.466 (96 h) for UNC8153, 1.752 (48 h) and 0.959 (96 h) for Fexofenadine hydrochloride, and 1.780 (48 h) and 1.573 (96 h) for novobiocin. Two‐way ANOVA was performed to evaluate the effects of compound concentration and exposure time on parasite growth inhibition. A significant main effect of exposure time was observed for UNC8153 (95% CI: 2.79–11.30; *p* = 0.0051), Fexofenadine hydrochloride (95% CI: 6.84–28.66; *p* = 0.0086), and novobiocin (95% CI: 3.91–19.40; *p* = 0.0118). Data points represent individual values obtained from two independent biological experiments (*n* = 2). Concentration is presented as log_10_ (μM).

To evaluate the antiplasmodial efficacy of UNC8153, Fexofenadine hydrochloride, and novobiocin, dose–response inhibition assays were conducted against *Plasmodium falciparum* (3D7 strain) over two time points: 48 h and 96 h post‐treatment. Parasite growth inhibition was measured, and IC_50_ (Half‐Maximal Inhibitory Concentration) values were calculated from non‐linear regression of the dose–response curves using GraphPad Prism (Figure 7). UNC8153 exhibited potent and time‐dependent antiplasmodial activity. At 48 h post‐treatment, the IC_50_ was 1.14 μM, which decreased significantly to 0.34 μM at 96 h, indicating a 3.4‐fold increase in efficiency during the 2nd life cycle. Fexofenadine hydrochloride has a relatively high IC_50_ of 30.25 μM at 48 h, but this was reduced to 14.23 μM at 96 h, indicating approximately a 2.1‐fold increase in effectiveness in the 2nd life cycle. Novobiocin, the known PfGyrB inhibitor, shows an IC_50_ of 13.83 μM at 48 h, which decreased to 8.38 μM at 96 h, indicating enhanced inhibitory activity during prolonged exposure. Notably, our tested drugs, UNC8153 and Fexofenadine hydrochloride, show a similar exposure‐dependent inhibitory trend compared with novobiocin. Interestingly, our identified molecule UNC8153 shows ~12‐fold and ~25‐fold higher efficiency than the validated PfGyrB inhibitor Novobiocin at the 1st life cycle (48 h) and 2nd life cycle (96 h), respectively, indicating greater antiplasmodial potency under the tested experimental conditions.

### 3.6 Effect of Drug Treatment on Parasite Morphology

**FIGURE 8:**
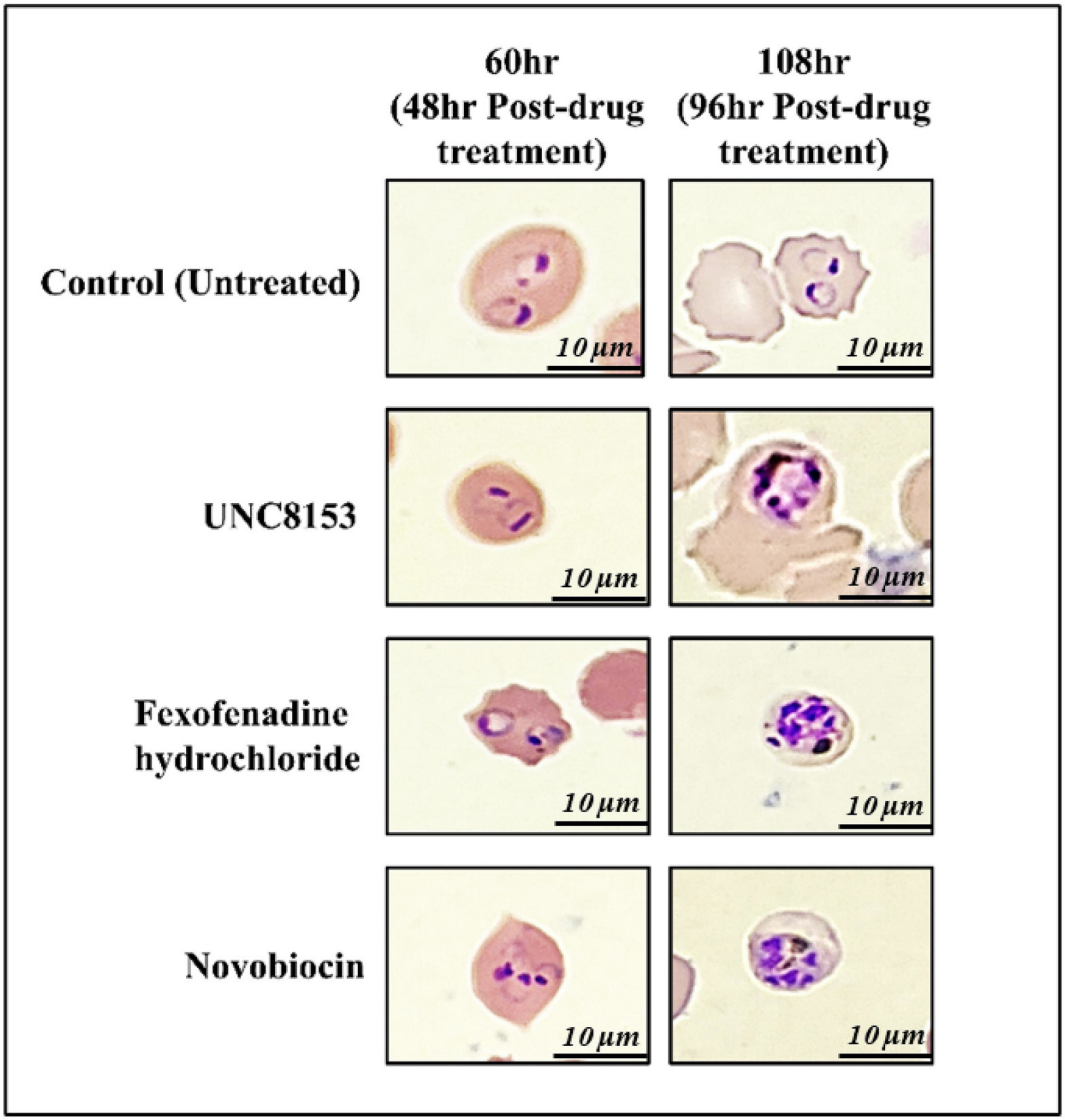
Representative Giemsa‐stained blood smears showing the morphology of *Plasmodium falciparum* (3D7) following treatment with UNC8153, Fexofenadine hydrochloride, and novobiocin. Parasite morphology was examined at 60 h (corresponding to 48 h post‐drug treatment) and 108 h (corresponding to 96 h post‐drug treatment) to evaluate parasite development under continuous compound exposure. Untreated parasites served as the control. Scale bar = 10 μm.

To investigate the effect of tested drugs on parasite morphology, the Giemsa‐stained blood smears were prepared at 60 h (48 h post‐drug treatment) and 108 h (96 h post‐drug treatment) to assess parasite development (Figure 8). It was observed that in the untreated control group, parasites progressed normally through both the 1st and 2nd intra‐erythrocytic cycles. However, treatment with UNC8153, Fexofenadine hydrochloride, and the known control drug novobiocin resulted in impaired parasite development, particularly evident at 108 h (2nd life cycle). Parasites exposed to these compounds exhibited arrested or abnormal morphology, indicating failure to complete the second intraerythrocytic life, consistent with impaired parasite development during prolonged compound exposure.

### 3.7 Cell Cytotoxicity Assay Reveals Limited Cytotoxicity of UNC8153 and Fexofenadine Hydrochloride Under the Tested Conditions

**FIGURE 9:**
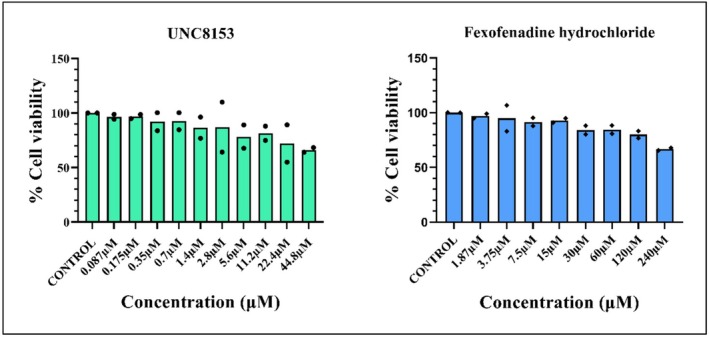
Cytotoxicity assessment of (A) UNC8153 and (B) Fexofenadine hydrochloride in HEK‐293 cells using the MTT assay. Cells were treated with increasing concentrations of the compounds, and cell viability was determined relative to untreated control cells. Data points represent individual values obtained from two independent biological experiments (*n* = 2).

To assess the cytotoxic potential of the tested compounds on human cells, an MTT assay was performed using the HEK‐293 (human embryonic kidney) cell line in two independent biological experiments with triplicate measurements. Cells were treated with increasing concentrations of UNC8153 and Fexofenadine hydrochloride for 24 h, after which cell viability was measured using an ELISA plate reader. As shown in Figure 9, it was observed that more than 60% of cells are viable at the highest concentration of UNC8153 (44.8 μM) and Fexofenadine hydrochloride (240 μM), indicating that both compounds have limited cytotoxicity under the tested conditions to human HEK‐293 cells. This inference is mainly based on the preliminary in vitro cytotoxicity data, while further in vivo and clinical safety studies might give better insights into their safety profile.

### 3.8 Structural Similarity Study Reveals UNC8153 and Fexofenadine Hydrochloride Are Distinct From Existing Antimalarial Drugs

**FIGURE 10:**
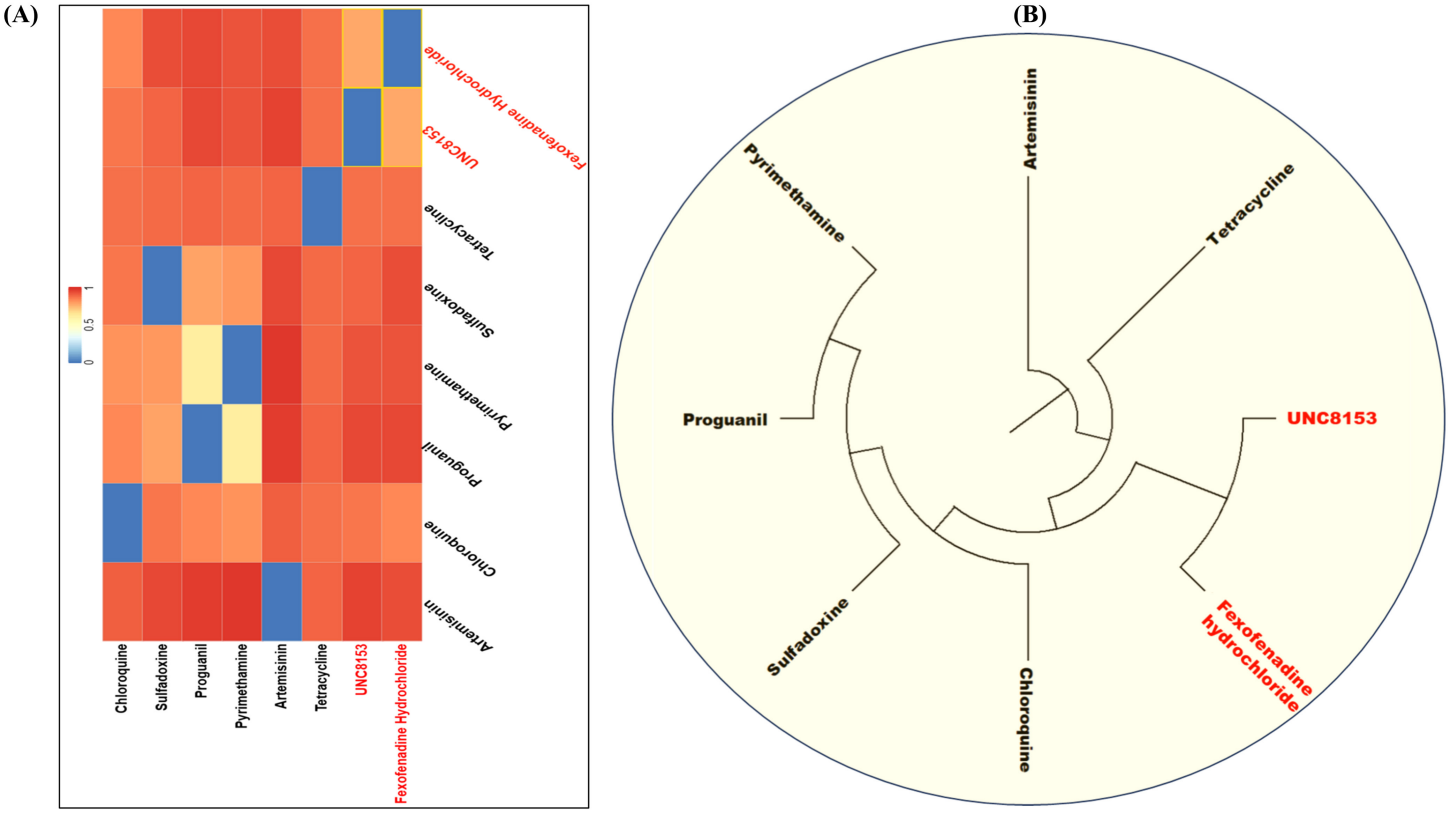
Structural similarity analysis of UNC8153, Fexofenadine hydrochloride, and selected antimalarial drugs. (A) Heatmap showing pairwise chemical similarity relationships based on the calculated distance matrix scores. Color intensity ranges from blue (lower distance score; higher structural similarity) to red (higher distance score; lower structural similarity). (B) Hierarchical clustering dendrogram generated from the distance matrix, illustrating the relative structural relationships among the analyzed compounds. The analysis indicates that UNC8153 and Fexofenadine hydrochloride are structurally distinct from currently used antimalarial drugs.

Structural novelty is a critical factor in overcoming drug resistance. To assess whether the identified inhibitors, UNC8153 and Fexofenadine hydrochloride, are structurally distinct from currently used antimalarial drugs, we conducted a similarity analysis using the Tanimoto coefficient and distance matrix scores. The resulting heat maps (Figure 10A) and phylogenetic trees (Figure 10B) indicate that both test compounds exhibit significant structural differences from known antimalarials, with distance matrix scores exceeding 0.5. UNC8153 and Fexofenadine hydrochloride are grouped, reflecting their structural similarity (Figure 10B). Altogether, the identified test compounds with antiplasmodial activity are structurally distinct from existing antimalarial drugs. This structural divergence suggests structural divergence from currently used antimalarial scaffolds, although dedicated resistance‐selection studies are required to evaluate cross‐resistance potential.

### 3.9 UNC8153 Inhibits Artemisinin‐Resistant *Plasmodium falciparum* (C580Y)

**FIGURE 11:**
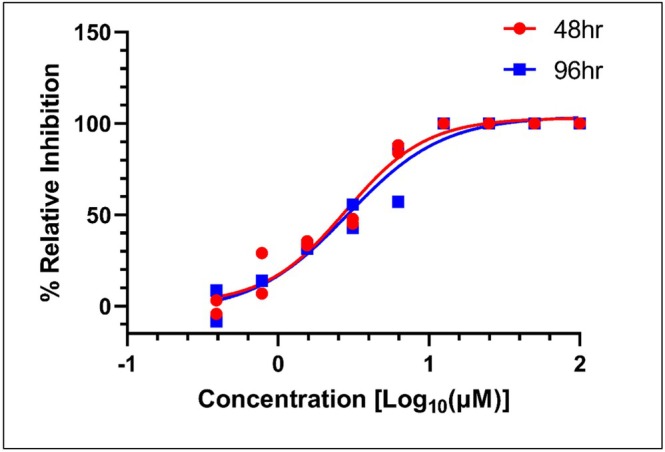
Dose‐dependent antiplasmodial activity of UNC8153 against the artemisinin‐resistant *Plasmodium falciparum* C580Y strain. (A) Dose–response curves showing percentage parasite growth inhibition following 48 h (red circles) and 96 h (blue squares) exposure to UNC8153. Curves were generated using a four‐parameter variable‐slope nonlinear regression model in GraphPad Prism for IC_50_ determination. Hill slope values obtained from the fitted models were 1.619 for 48 h and 1.377 for 96 h. Two‐way ANOVA was performed to evaluate the effects of compound concentration and exposure time on parasite growth inhibition. No statistically significant main effect of exposure time was observed between the 48 h and 96 h treatment groups (*p* = 0.3271). Data points represent results from two independent biological experiments (*n* = 2), and concentration is presented as log_10_ (μM).

To evaluate whether the potent compound UNC8153 is effective against artemisinin‐resistant *Plasmodium falciparum* (C580Y), we treated the parasites with varying concentrations of UNC8153. The parasitaemia was measured at both 48 and 96 h of drug treatment. Our results show that UNC8153 inhibits the artemisinin‐resistant *P. falciparum* (C580Y) with an IC_50_ value of 2.8 μM at 48 h and 2.9 μM at 96 h, indicating similar efficacy across both the first and second parasite life cycles (Figure 11). Furthermore, we calculated the Resistance Index (RI) as the ratio of the IC_50_ value of a compound against a drug‐resistant strain to its IC_50_ against a drug‐sensitive strain for UNC8153, where an RI > 10 typically indicates high‐level drug resistance (White 2004). The RI value was calculated as 2.46 at 48 h for UNC8153, suggesting a lower potential for cross‐resistance at 48 h. These findings are consistent with the structural similarity analysis, which also indicated that these compounds are distinct from currently used antimalarials. However, we observed that UNC8153 did not exhibit enhanced inhibitory activity following prolonged exposure and showed an increased RI value of 8.52 at 96 h, suggesting future studies to explore the mechanism of action of UNC8153 in an artemisinin‐resistant strain.

## 4 Discussion

The world is facing major challenges in combating the increase in antimalarial drug resistance, particularly in the malaria‐endemic regions (Venkatesan 2025). In this scenario, there is an urgent need for novel drugs that can overcome the existing challenge of rising resistance issues. When we evaluate the solution, it is important to keep in mind that the identified drug should have a novel mode of action and be structurally distinct from existing antimalarials to avoid cross‐resistance (Tang et al. 2020). In this study, we have proposed an integrative approach to target the PfGyrB protein of the apicoplast, as it is known for being a validated drug target for antibiotics such as Novobiocin. Unlike its bacterial homologs, PfGyrB exhibits lower sequence identity (~38%) but retains conserved ATPase motifs essential for its DNA supercoiling function (Dar et al. 2007). This unique divergence provides opportunities for selective inhibition while minimizing host toxicity.

We have curated a compound library that is structurally 80% similar to Novobiocin and screened against the PfGyrB protein to find better hits comparable to Novobiocin. Many researchers have adopted this virtual screening approach and successfully identified the hits, which were further found to be lead molecules in several infectious diseases, including malaria (Godara et al. 2024; Naik et al. 2025, 2024, 2020, 2022; Njogu et al. 2016). Recently, we employed a similar target‐based drug discovery approach and successfully identified novel antimalarial chemical entities that specifically inhibit the ssDNA binding property of PfSSB, a protein indispensable for apicoplast genome replication (Naik et al. 2025).

So, we thoroughly analyzed the binding potential of the compounds inside the ATPase domain of PfGyrB. The molecular insights of the top‐ranked docked complexes revealed that the hit compounds perfectly bound with the ATPase domain, interacting with the key conserved and crucial residues like GLU159, ASN163, and HIS282 (Figure 3), consistent with the catalytic importance of these residues highlighted in earlier biochemical characterizations (Ram et al. 2007). These compounds again show stable conformational changes, which justify their energetically favorable binding to PfGyrB in a real‐time virtual dynamic environment (Figure 4).

The BLI analysis demonstrated detectable binding interactions between UNC8153, Fexofenadine hydrochloride, Novobiocin, and PfGyrB under the tested experimental conditions. However, in contrast to Fexofenadine hydrochloride and Novobiocin, the sensorgram profile observed for UNC8153 displayed a rapid signal increase during the association phase, followed by an abrupt signal decrease during the early dissociation phase. This behavior may potentially reflect bulk refractive index effects, rapid dissociation kinetics, and/or nonspecific interactions associated with the assay conditions or compound‐specific properties. Therefore, additional biophysical and kinetic studies will be required to further clarify the nature of the UNC8153‐PfGyrB interaction.

Previous studies reported that PfGyrB exhibits DNA‐stimulated ATPase activity, distinct from its bacterial counterpart (Dar et al. 2009). Our DNA‐stimulated ATPase inhibition assays confirmed that UNC8153 competitively inhibits ATP hydrolysis with potency comparable to novobiocin. This result builds directly on earlier biochemical characterizations of PfGyrB. The calculated *K*_i_ of Novobiocin (0.320 ± 0.023 μM) is close to the previously reported *K*_i_ in the PfGyrB ATPase inhibition assay (Ram et al. 2007). However, the 2nd top hit, Fexofenadine hydrochloride, showed comparable PfGyrB ATPase inhibition activity to UNC8153 and Novobiocin.

The antibiotic novobiocin was previously reported to inhibit PfGyrB ATPase activity and display modest antiplasmodial activity with 80% parasitemia clearance at 30 μM (Ram et al. 2007). In the parasite growth inhibition study, we observed that UNC8153 demonstrated ~25‐fold higher potency than novobiocin in the second parasite life cycle. The enhanced inhibitory activity in the 2nd life cycle observed following prolonged exposure to UNC8153 parallels findings from studies with apicoplast inhibitors such as clindamycin and doxycycline (Dahl and Rosenthal 2007). These antibiotics disrupt apicoplast translation, leading to impaired survival only in subsequent life cycles. Our morphological analysis, revealing defective parasite development in the second cycle, is consistent with this mode of action (Figure 8). The convergence of our results with these earlier observations strengthens the argument that UNC8153 specifically targets the apicoplast and not off‐target parasite processes (Dahl and Rosenthal 2007). In addition, inclusion of established antimalarial reference compounds such as chloroquine, artemisinin, and doxycycline in future comparative assays would further strengthen interpretation of compound potency, stage‐specific activity, and delayed inhibitory effects relative to clinically relevant antimalarial classes.

The emergence of resistance to existing antimalarial drugs and the development of cross‐resistance to newly discovered compounds pose significant challenges to malaria control and eradication efforts (Ashley et al. 2014). Here, our structural similarity analysis revealed that both UNC8153 and Fexofenadine hydrochloride are structurally distinct from existing antimalarial drugs, suggesting that they could yield new scaffold antimalarial compounds. This structural divergence is particularly important because it reduces the likelihood of pre‐existing resistance. Structural novelty plays a crucial role in overcoming resistance by evading target‐specific mutations or efflux mechanisms associated with known drug classes (Bopp et al. 2013; Burrows et al. 2013). Unlike doxycycline and clindamycin, which already face declining efficacy in certain malaria‐endemic regions (Barman and Goswami 2025), UNC8153's novelty suggests that resistance may emerge more slowly. Importantly, UNC8153 retained efficacy against artemisinin‐resistant parasites (C580Y), with only a modest resistance index (RI ~2.46) at 48 h, in contrast to artemisinin partner drugs like piperaquine, which exhibit high cross‐resistance (Watson et al. 2022). Interestingly, UNC8153 showed an increased RI value of ~8.20 at 96 h against the artemisinin‐resistant C580Y strain, indicating a more complex response under prolonged exposure conditions. Previous studies on apicoplast‐targeting compounds such as doxycycline have shown that concentration‐dependent effects can influence the balance between delayed second‐cycle inhibition and more immediate parasite growth inhibition (Okada et al. 2020). Therefore, the observed response profile of UNC8153 may reflect additional mechanistic complexity beyond a classical delayed‐death phenotype, potentially involving concentration‐dependent or off‐target effects. Further mechanistic studies, including compound washout and IPP rescue assays, will be necessary to clarify the underlying mode of action.

Host toxicity remains a major limitation in antimalarial drug development. Earlier cytotoxicity studies showed that novobiocin had limited selectivity, reducing its appeal as a candidate (Sharma et al. 2025). In contrast, our findings show that UNC8153 was minimally toxic to human HEK‐293 cells even at the highest dose of 44.8 μM, providing preliminary evidence of favorable in vitro selectivity under the tested experimental conditions. Although preliminary cytotoxicity assessment in HEK‐293 cells suggested limited toxicity under the tested conditions, potential inhibitory effects on human topoisomerases or related host enzymes were not experimentally evaluated in the current study and require further investigation for comprehensive safety assessment. Additionally, future in vivo toxicity and pharmacokinetic studies will be necessary to better evaluate the overall safety profile of these compounds.

While our study substantially extends previous investigations targeting PfGyrB (M. A. Dar et al. 2007; Ram et al. 2007), several important limitations should be acknowledged. The absence of an experimentally resolved crystallographic structure for PfGyrB necessitated reliance on homology modeling. Although the generated model was supported by QMEANDisCo, local QMEAN, and Ramachandran analyses, structural inaccuracies within flexible regions may influence ligand positioning and interaction prediction (Benkert et al. 2009). In addition, the present study remains limited to in vitro and computational analyses. Therefore, future in vivo efficacy, pharmacokinetic, and toxicity studies will be required to further evaluate the therapeutic potential of UNC8153. Although UNC8153 demonstrated enhanced antiplasmodial activity relative to novobiocin under the tested experimental conditions, the relatively modest reduction in IC_50_ observed between 48 h and 96 h exposure was substantially smaller than the delayed inhibitory effects reported for established apicoplast‐targeting compounds such as doxycycline (Rosenthal 2016; Tan et al. 2011). Furthermore, because comparable PfGyrB interaction profiles were observed in the BLI and ATPase assays, the stronger antiplasmodial activity of UNC8153 may potentially involve additional parasite targets beyond PfGyrB. In this context, possible off‐target interactions with proteins such as PfRuvB, which is also a target of Novobiocin, cannot currently be excluded and require further mechanistic investigation (Ahmad et al. 2015). Additionally, canonical delayed‐death validation assays, including compound washout and IPP rescue experiments, were not performed in the current study. Therefore, the observed increase in inhibitory activity at prolonged exposure time points is interpreted as delayed inhibitory effects rather than a classical delayed‐death phenotype. Future studies evaluating intracellular target engagement, resistance selection, and combination therapy with fast‐acting antimalarials may further clarify the therapeutic potential of UNC8153 against drug‐resistant malaria.

## 5 Conclusion

This study identified UNC8153 as a structurally distinct new antiplasmodial scaffold associated with inhibitory activity against *Plasmodium falciparum* and biochemical interaction with PfGyrB. Although UNC8153 demonstrated greater antiplasmodial activity than novobiocin under the tested experimental conditions and retained activity against an artemisinin‐resistant parasite line, the precise mechanism underlying its antiplasmodial effects remains to be fully elucidated. Furthermore, the observed exposure‐dependent inhibition should be interpreted cautiously because canonical delayed‐death validation assays were not performed. Taken together, the findings support further investigation of PfGyrB‐associated inhibitors as potential antimalarial agents and establish UNC8153 as a candidate for additional mechanistic, pharmacokinetic, and in vivo studies. Future work involving intracellular target validation, resistance profiling, and efficacy assessment in animal models will be essential to determine its suitability for antimalarial drug development.

## Author Contributions

**Biswajit Naik:** conceptualization, investigation, methodology, validation, visualization, writing – review and editing, writing – original draft, software, formal analysis, data curation. **Chandi C. Mandal:** formal analysis, supervision, resources. **Dhaneswar Prusty:** investigation, conceptualization, funding acquisition, writing – review and editing, resources, supervision, project administration, formal analysis. **Gajendra Mohan Baldodiya:** methodology, data curation, validation. **Welka Sahu:** methodology, data curation, validation. **Jeong Ho Hwang:** supervision, investigation, resources. **Guneswar Sethi:** methodology, data curation, software. **K. Sony Reddy:** supervision, formal analysis, resources, investigation. **Cherish Prashar:** methodology, data curation, validation. **Jyoti Poswal:** methodology, data curation, validation. **Kailash C. Panday:** supervision, investigation, resources.

## Funding

The authors gratefully acknowledge the Indian Council of Medical Research, New Delhi, India, for providing financial support to Dr. Dhaneswar Prusty in the form of a research grant (EMDR/IG/10‐2023‐0000879).

## Disclosure

Declaration of generative AI in scientific writing: All the authors declare that they have not utilized AI to create or analyze this scientific work.

## Ethics Statement

The *P. falciparum* culture experiments were carried out using O^+^ human erythrocytes collected from voluntary donors after obtaining written informed consent. All sample collection and processing procedures were conducted in compliance with the protocols approved by the Institutional Ethics Committee (KIIT/KIMS/IEC/167/2021). The study was performed in strict accordance with the ethical principles laid down in the World Medical Association's Declaration of Helsinki.

## Conflicts of Interest

The authors declare no conflicts of interest.

## Supporting information


**Data S1:** cbdd70364‐sup‐0001‐DataS1.pdb.

**Figure S1:** SDS‐PAGE analysis of recombinant PfGyrB expression and purification. Proteins were resolved on a 10% SDS‐PAGE gel and visualized by Coomassie Brilliant Blue staining. Lane Ladder, pre‐stained protein molecular weight marker (10–250 kDa); Lane WCL, whole‐cell lysate; Lane TSP, total soluble protein; Lane FT, flow‐through fraction; Lane wash and Lane elution fractions containing recombinant PfGyrB. The predominant band corresponding to the expected molecular weight of PfGyrB is indicated by the arrow.
**Table S1:** Raw data of ATPase inhibition assay representing Initial Rate (*V*_0_) μmol/min at different concentrations of ATP from two independent experiments.

## Data Availability

All data generated or analyzed during this study are included in this published article and it's Supporting Information. The refined PfGyrB homology model (.pdb) is provided as a Supporting Information. Further information is available from the corresponding author upon reasonable request.
