# Supplementary material for: Discovery of Potent Antimalarial Agents Targeting Plasmodium falciparum DNA Gyrase B by Integrating Computational and Experimental Approaches
Source: Chem Biol Drug Des. 2026 Jul 21;108(1):e70364. doi: 10.1111/cbdd.70364 (PMC13389332; doi:10.1111/cbdd.70364)
Supplement: Supplementary file 2 — Figure S1: SDS‐PAGE analysis of recombinant PfGyrB expression and purification. Proteins were resolved on a 10% SDS‐PAGE gel and visualized by Coomassie Brilliant Blue staining. Lane Ladder, pre‐stained protein molecular weight marker (10–250 kDa); Lane WCL, whole‐cell lysate; Lane TSP, total soluble protein; Lane FT, flow‐through fraction; Lane wash and Lane elution fractions containing recombinant PfGyrB. The predominant band corresponding to the expected molecular weight of PfGyrB is indicated by the arrow. Table S1: Raw data of ATPase inhibition assay representing Initial Rate (V 0) μmol/min at different concentrations of ATP from two independent experiments. [file CBDD-108-e70364-s002.docx]

**Discovery of Potent Antimalarial Agents Targeting *Plasmodium falciparum* DNA Gyrase B by Integrating Computational and Experimental Approaches**

**Biswajit Naik^1^, Welka Sahu^2^, Guneswar Sethi^3^, Cherish Prashar^4,5^, Gajendra Mohan Baldodiya^1^, Jyoti Poswal^1^, Chandi C mandal^1^, Jeong Ho Hwang^6^, Kailash C Panday^4,5^, K Sony Reddy^2^, Dhaneswar Prusty^1*^**

^1^Department of Biochemistry, School of Life Sciences, Central University of Rajasthan,

Bandarsindri, Ajmer, Rajasthan, 305817, India

^2^School of Biotechnology, Kalinga Institute of Industrial Technology, Bhubaneswar, Odisha, 751024, India

^3^Center for Large Animals Convergence Research, Korea Institute of Toxicology, Jeongeup-si, Jeollabuk-do, 56212, Korea

^4^ICMR-National Institute of Malaria, Sector 8, Dwarka, New Delhi, 110077, India

^5^Academy of Scientific and Innovative Research (AcSIR), Gaziabad, 201002, India

^6^Division of Advanced Predictive Research, Center for Bio-Signal Research, Korea Institute of Toxicology, Daejeon, 34114, Republic of Korea

***Corresponding author**

Dhaneswar Prusty

Department of Biochemistry

School of Life Sciences

Central University of Rajasthan

Bandarsindri, Ajmer, Rajasthan-305817, India

Email: [dhaneswarprusty@curaj.ac.in](mailto:dhaneswarprusty@curaj.ac.in)

**Supplementary Figure 1**: SDS-PAGE analysis of recombinant PfGyrB expression and purification. Proteins were resolved on a 10% SDS-PAGE gel and visualized by Coomassie Brilliant Blue staining. Lane Ladder, pre-stained protein molecular weight marker (10-250 kDa); Lane WCL, whole-cell lysate; Lane TSP, total soluble protein; Lane FT, flow-through fraction; Lane wash and Lane elution fractions containing recombinant PfGyrB. The predominant band corresponding to the expected molecular weight of PfGyrB is indicated by the arrow.


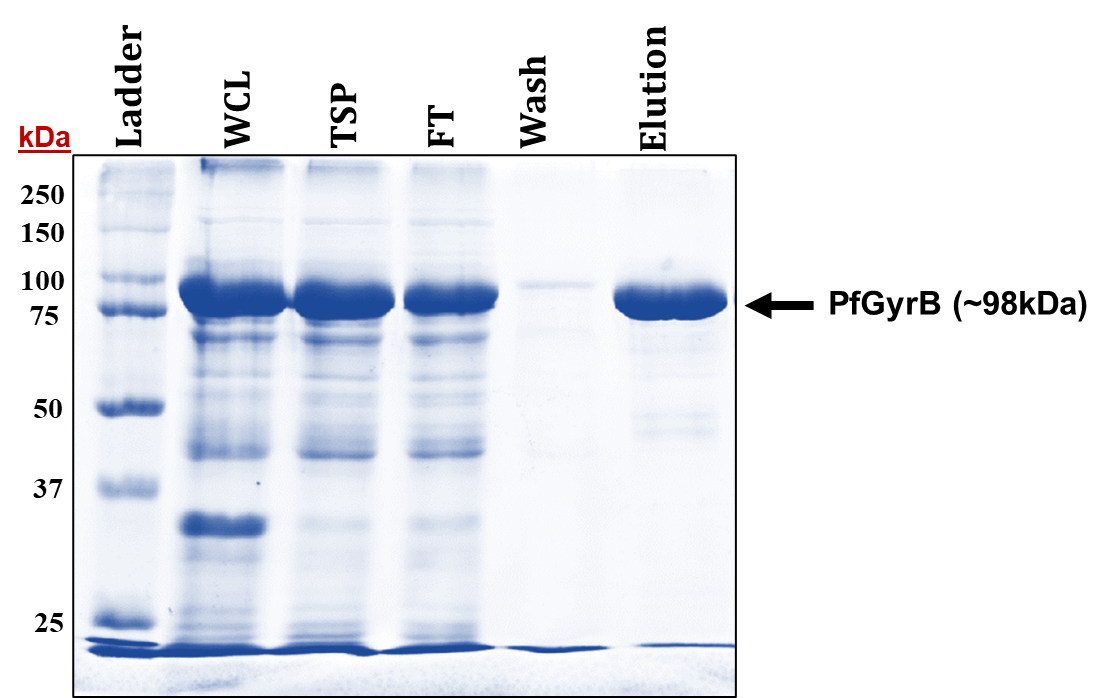


**Supplementary Table 1**: Raw data of ATPase inhibition assay representing Initial Rate (V_0_) μmol/min at different concentrations of ATP from two independent experiments.

|  | **Initial Rate (V_0_) μmol/min** | | | | | | | |
| --- | --- | --- | --- | --- | --- | --- | --- | --- |
| **ATP conc. (mM)** | **Control** | | **Novobiocin (10µM)** | | **UNC8153**  **(10µM)** | | **Fexofenadine hydrochloride (10µM)** | |
|  | **EXPT 1** | **EXPT 2** | **EXPT 1** | **EXPT 2** | **EXPT 1** | **EXPT 2** | **EXPT 1** | **EXPT 2** |
| **0** | 0.00 | 0.00 | 0.00 | 0.00 | 0.00 | 0.00 | 0.00 | 0.00 |
| **0.05** | 0.13 | 0.08 | 0.00 | 0.00 | 0.01 | 0.00 | 0.11 | 0.11 |
| **0.1** | 0.21 | 0.17 | 0.01 | 0.01 | 0.04 | 0.03 | 0.11 | 0.12 |
| **0.15** | 0.28 | 0.21 | 0.02 | 0.01 | 0.05 | 0.03 | 0.12 | 0.13 |
| **0.2** | 0.34 | 0.28 | 0.04 | 0.02 | 0.08 | 0.06 | 0.14 | 0.12 |
| **0.25** | 0.27 | 0.22 | 0.04 | 0.02 | 0.07 | 0.04 | 0.15 | 0.14 |
| **0.5** | 0.40 | 0.35 | 0.07 | 0.05 | 0.10 | 0.08 | 0.22 | 0.21 |
| **0.75** | 0.42 | 0.36 | 0.09 | 0.06 | 0.11 | 0.08 | 0.2 | 0.19 |
| **1** | 0.47 | 0.44 | 0.12 | 0.05 | 0.13 | 0.11 | 0.27 | 0.26 |
| **1.5** | 0.39 | 0.29 | 0.19 | 0.13 | 0.17 | 0.14 | 0.3 | 0.27 |
| **2** | 0.31 | 0.35 | 0.15 | 0.10 | 0.11 | 0.11 | 0.34 | 0.33 |
